# Design and Development of In-House Multichannel Applicator for HDR Vaginal Brachytherapy and Dosimetric Comparision with Single Channel Applicator

**DOI:** 10.31557/APJCP.2019.20.12.3805

**Published:** 2019

**Authors:** Kesavan Govindaraj, Senthilkumar Shanmugam, Sowmiya Sampathrajan

**Affiliations:** 1 *Department of Radiotherapy, Vadamalayan Hospitals Integrated Cancer Centre, *; 3 *Department of Radiotherapy, Madurai Medical College and Govt. Rajaji Hospital, Madurai, *; 2 *Research and Development Centre, Bharathiar University, Coimbatore, India. *

**Keywords:** Endometrial carcinoma, in-house multichannel applicator, single channel applicator, brachytherapy

## Abstract

**Introduction:**

Endometrial cancer (EC) is a leading cause of female cancer in developed countries. The total abdominal hysterectomy and bilateral salpingo-oophorectomy with pelvic lymph node dissection is the primary management of endometrial cancer. Afterwards vaginal brachytherapy can be preferred either alone or combined with external beam radiotherapy to avoid the vault recurrence. This study was to assess the in-house multichannel applicator for HDR vaginal brachytherapy and dosimetric compression with the single channel applicator through DVHs. The main objective of this study was to design and development of an in-house multichannel applicator for HDR vaginal brachytherapy and dosimetric compression with the single channel applicator through DVHs.

**Materials and Methods:**

The multichannel applicator is a solid cylinder with 3 cm diameter, 13 cm length. It has a central channel surrounded by eight channels with a periphery arrangement in a single circle. We randomly selected eleven patients with endometrial carcinoma and 7Gy/per fraction of HDR dose was prescribed to CTV. Retrospectively, two 3D inverse treatment plans were created for each patient, with single channel loading and multichannel loading and the dose distribution of both plans could be compared. CTV coverage, rectum and bladder doses were compared.

**Results:**

The DVH analysis showed statistically significant difference between single and multichannel plan, that is for D95 of CTV (p=0.008), D100 of CTV (p=0.004) and 2cc of CTV (p=0.003). The p value for 1cc, 2cc and V70 are 0.003, 0.003 and 0.003 for rectum. On the other hand, bladder DVHs showed large difference between single and multichannel plan yet it is not statistically significant, and the p values for 1cc, 2cc and V 80 are 0.012, 0.009 and 0.225.

**Conclusion:**

The authors conclude that in house multichannel applicator with 3D inverse treatment planning techniques improves the dosimetric advantage over single channel applicators.

## Introduction

Most common gynecological malignancies are cervix and uterine carcinomas. Endometrial cancer (EC) is a leading cause of female cancer in developed countries. It is the fifth most common cancer worldwide and eighth leading cause of death from cancer in women (Sebastià et al., 2017; Ahmedin et al., 2009). The total abdominal hysterectomy and bilateral salpingo-oophorectomy with pelvic lymph node dissection is the primary management of endometrial cancer. According to the stage and grade of the endometrial carcinoma adjuvant therapy is decided and after hysterectomy, adjuvant radiotherapy plays a crucial role in the treatment of these carcinomas (Herbert et al., 1996). Radiotherapy can be delivered with external beam radiation therapy (EBRT) and /or brachytherapy (Henry et al., 2004; Small et al., 2012). 

Generally the aim of brachytherapy is to insert radioactive sources into endometrium or vagina (post-hysterectomy) to deliver prescribed dose to primary target volume and minimize radiation to critical organs. Traditionally brachytherapy dose was delivered by the low dose rate (LDR) application technique and medium dose rate (MDR) using Radium-223 and Cs-137 sources. A later LDR and MDR techniques was replaced by high dose rate (HDR) because of short treatment time, less hospital stay for patient and also possibility of optimization in planning (Sanjay et al., 2009). Nowadays Ir-192 is the mainly used source for high dose rate remote after loading brachytherapy for intra vaginal applicators (Nazarnejad et al., 2012).

The major concern of high-dose-rate (HDR) brachytherapy was late radiation reactions particularly for the young and sexually active patients. Similarly there is high possibility of radiation toxicity risk for surrounding normal organ such as bladder and rectum.

Single-channel vaginal cylinders are more frequently used to treat the vaginal cuff, but they are limited in their ability to sculpt dose away from organs at risk (OARs). Multichannel applicators, through modulation of dwell times in various positions along the channels, improve the ability to optimize target coverage and to minimize dose to OARs (Yasir et al., 2014). 

The main objective of this study was to design and develop an in-house multichannel applicator for HDR vaginal brachytherapy and dosimetric comparison with the single channel applicator with dose volume histograms (DVHs).

## Materials and Methods

*Applicator design*

**Figure 1:**
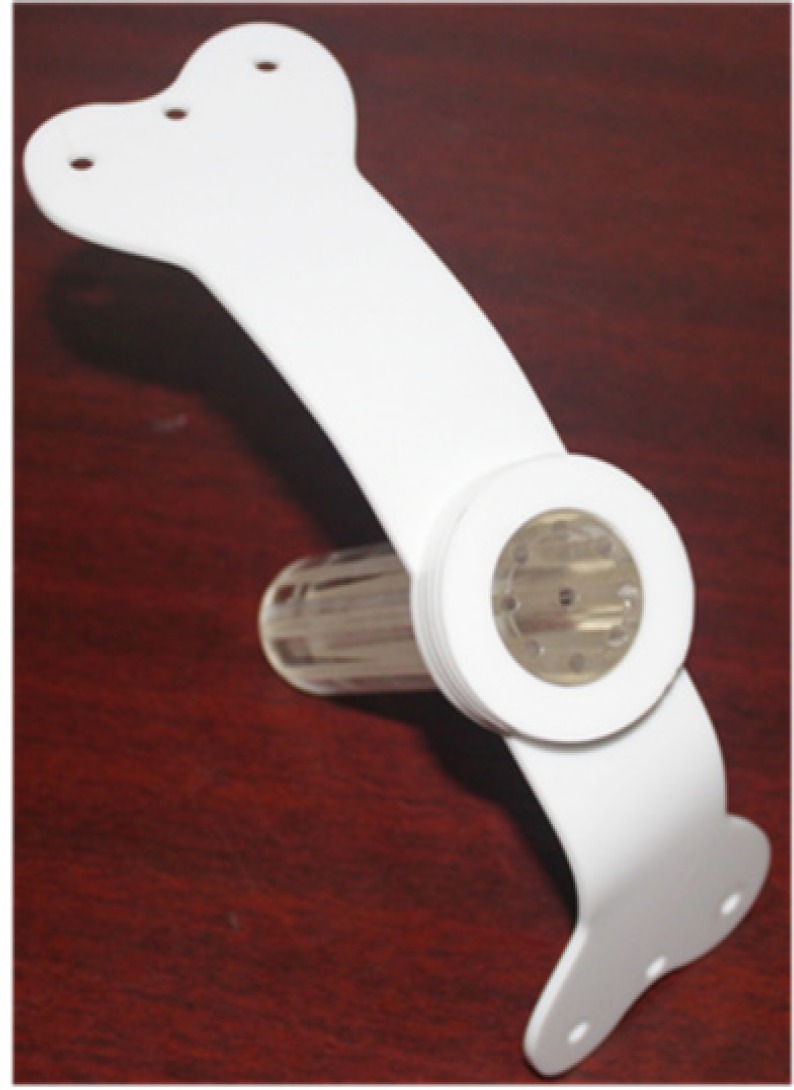
Design of Multichannel Applicator

**Figure 2:**
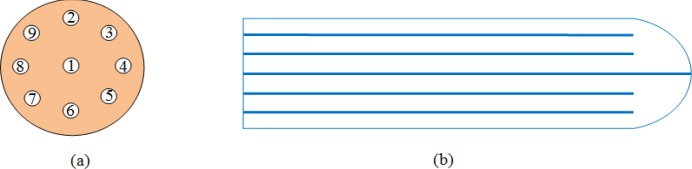
a) Axial view of multichannel applicator with mention the channel number; b) Sagittal View of multichannel applicator

Computer Aided Design (CAD) software (Autodesk, Inc., San Francisco, CA, USA) was used to create a design file for a desired applicator shape, which is directly read by a CNC machine and precise hole drilling in poly methyl methacrylate (PMMA). The multichannel applicator is a solid cylinder with 3 cm diameter, 13 cm length. The apical tip of the applicator is hemispherical dome shaped. It has one channel in the centre and eight are in the periphery arranged in a single circle, which is 0.5 cm from the surface of cylinder. The in-house multichannel applicator is sterilizable and suitable for multiple uses. The in-house multichannel applicator is show in the Figure 1. There is an external fixator at the end of the applicator made up of plastic and it was slightly curved to fit to patient anatomy its help to application remain in fixed in patient during the course of the treatment. The axial view and Sagittal view of multichannel applicator is given in the Figure 2. 

*Patient Selection, Applicator Insertion and CT simulation*

We randomly selected 11 patients with endometrial carcinoma who have undergone radical hysterectomy. Each woman received a combination of external beam radiotherapy (EBRT) to treat regional lymph nodes and HDR brachytherapy to boost the vaginal cuff and the summarized patient characteristics given in Table 1. The prescribed EBRT dose for all patients was 45 Gy, to be delivered in 1.8 Gy daily fractions. The HDR boost delivered 7 Gy/fraction, for a total of 21 Gy. Patients were requested to evacuate the rectum and bladder just before the applicator insertion. In order to help delineating the bladder and rectum, a 20 cc of urographine contrast media was injected into the bladder (using Foley’s catheter) and a rectum catheter was inserted into the rectum.

Each patient underwent CT-scan with commercially available single channel applicator, planning done and treatment was delivered. The multichannel applicator was inserted for dosimetric comparison purpose with central channel loading applicator.

The applicator was covered with a lubricated condom and inserted into the patient on dorsal lithotomy position. For smooth insertion mild pressure applied superiorly on apex which also helps cylinder remains in physical contact with the vaginal apex. During the procedure there is no need of local anesthesia or sedation. 

In order to maintain the device at a consistent position for accurate treatment, the cylinder is rotated to orient the center of channel 2 anteriorly; the knee rest was placed below the patient leg in order to avoid movement of patient.

Computerized tomography images without intravenous contrast were acquired using a CT scanner (GE Discovery IQ), with 2 mm slice intervals from the iliac crest to the distal end of the applicator. The images were transferred to Eclipse TPS, by DICOM RT network. 

*Patient Contouring, Planning and Plan Evaluation*

After importing the image, the body was contoured automatically. The rectum was contoured from 1 cm above anus, up to the recto-sigmoid junction. The bladder was contoured as an entire organ including the outer wall of the bladder upto the beginning of urethra. The applicators were reconstructed by selecting on each slice of CT images. The CTV was contoured in the axial slice of image, 5 cm length measured from the tip of the applicator and 5 mm expansion from applicator surface and cropped from rectum and bladder. 

Planning was performed by Varian Brach vision Treatment planning system (TPS) and the treatment was executed by Varian HDR Gamma Med IX (Varian Medical Systems, Palo Alto, CA, USA). Generally constraints for target and critical structures required as an input for volumetric optimization (VO). 7 Gy/per fraction HDR dose was prescribed to CTV. Dose constraints and all other planning parameters were kept same for single channel and multichannel approach. 

Two plans were created for each patient, with single channel loading and multichannel loadings such that dose distribution of both plans could be compared. The dwell positions were evenly spaced to cover the CTV. The standard loading pattern of source and dwell positions were fed into the planning system to generate a uniform dose distribution on CTV around the cylinder surfaces. In order to reduce dose bladder and rectum dose, the Channel 2 and 6 was not loaded because it was close to the bladder and rectum. Generally constraints for target and critical structures required as an input for volumetric optimization (VO). In order to compare the dose distribution of plans, all dose constraints and all other planning parameters were kept same for single channel and Multichannel approach. 

Table 2 shows that the dose constraints for volume optimization. Dose constraints for CTV target was 95% volume receive 100 % (7 Gy) dose and 100 % volume receive 95% prescribed dose (6.65 Gy). Dose constraints for 2 cc of rectum the volume to receive maximum 70% of the prescribed dose (4.9 Gy), similarly 2cc of bladder the volume to receive maximum 80% of the prescribed dose (5.6 Gy).

The dose calculation algorithm is based on the TG-43 formalism, as recommended by the American Association of Physicists in Medicine (AAPM) (Rivard et al., 2004). For plan evaluation, the Dose-volume histograms (DVHs) analyzed for target and OARs. The target coverage for 100%, 95% and 2cc (D_100_, D_95_ and 2cc respectively) of prescribed dose was noted. Dose received by the most exposed volumes 2 cm^3^ (D_2cc_) of CTV can be consider of high exposure of vaginal mucosa. The dose received by 1cc, 2cc, and V_80%_ of bladder was noted. The doses received by 1cc, 2cc and V_70%_ of rectum were noted. 

**Table 1:** Shows Summarized Patient Characteristics of the Sudy

Patients Characteristics	Value
Total number of Patients	11
Age - Median(Range) years	57 (38-73)
Stage	3
IA	5
IB	2
II	2
IIIC1	1
Grade	6
1	5
2	
Surgery	4
Simple hysterectomy	7
Radical hysterectomy	

**Table 2:** Dose Constraints for Volume Optimization of Single Channel and Multichannel

Organ	Volume	Dose constraints (Gy)	Priority (%)
CTV	95%	7.00	100
	100%	6.65	100
Rectum	2cc	4.90	100
Bladder	2cc	5.60	100

**Table 3:** Summarizes the Mean Dose and Standard Deviation Data of CTV, Bladder and Rectum of Single and Multichannel Application for Fractional Prescribed Dose of 7 Gy

	Single channel (mean)	Single channel (±SD)	Multichannel (mean)	Multichannel (±SD)	P value
CTV					
D_100_(%)	85.49	9.04	91.58	3.43	0.008
D_95_(%)	91.70	5.96	95.78	1.05	0.004
D_2cc_(Gy)	11.43	3.30	14.08	2.70	0.003
Rectum					
D_1cc_ ( Gy )	6.85	0.78	5.75	0.39	0.003
D_2cc_ ( Gy)	6.02	0.67	4.80	0.21	0.003
V_70%_ (CC)	7.46	3.80	4.11	1.92	0.003
Bladder					
D_1cc (_ Gy )	4.99	1.08	4.85	1.09	0.012
D_2cc_ ( Gy)	4.68	1.03	4.52	1.02	0.009
V_80%_ (CC)	0.65	0.78	0.51	0.64	0.225

**Figure 3:**
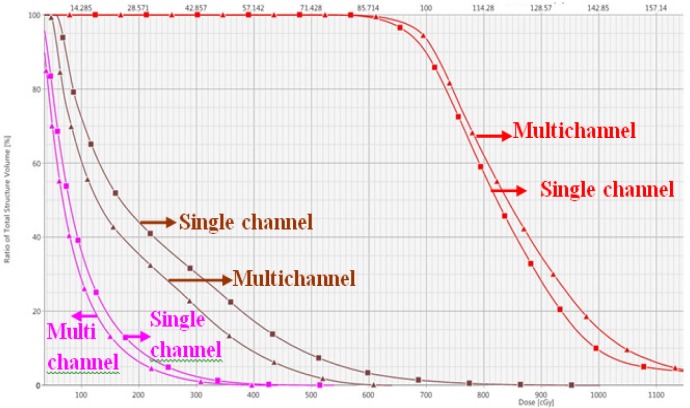
Dose-Volume Histogram Comparison between Single Channel (Squares) and Multichannel (Triangles) Approaches. Clinical target volume is displayed in red, rectum in brown and bladder in mangenta

**Figure 4:**
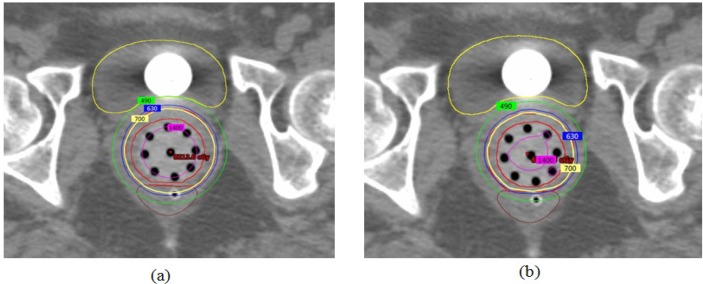
Dose Distribution Comparison between (a) Single Channel, (b) Multichannel. Isodose line of 7 Gy (prescribed dose) is displayed in yellow, 6.3 Gy (90% of prescribed dose) in Blue, 4.9 Gy (70% of prescribed dose) in Green and 14 Gy (200% of prescribed dose) in magenta

**Figure 5:**
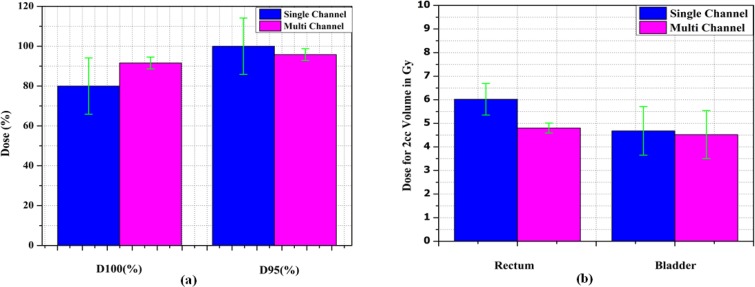
(a) and (b) Shows Mean and Standard Deviation (SD) of CTV D_100_(%), D_95_(%), D_2cc_(Gy) Bladder and D_2cc_(Gy) Rectum

## Results

A total of twenty two plan results were analyzed using DVHs which were generated for CTV, bladder and rectum of the 11 treated patients. It was performed Wilcoxon matched pairs test was performed for the statistical analysis and p value of < 0.05 was considered significant. Figure 3 shows that DVH comparison of single channel and multichannel applicator.

Totally 22 plans, were generated which contain 11 single channel plans and 11 multichannel plans. For multichannel all the dose constraints were fulfilled in 10 plans but in single channel none of them plan fulfilled all dose constraints. Clinical target volume dose coverage was attained for all plans of multichannel and 3 plans of single channel. For bladder of multichannel plan entire dose constrains were achieved and for rectum 10 plans were achieved. In single channel plan rectum dose constrains were not attained in 3 plans, but in bladder the entire dose constrains were fulfilled.

Table 3 shows summarizes the mean dose and standard deviation data of CTV, bladder and rectum of single and multichannel application for fractional prescribed dose of 7 Gy.

The DVH analysis showed statistically significant difference between single and multichannel plan, in terms of CTV coverage for D_100_ of CTV (p=0.008) and D_95_ of CTV (p=0.004). It clearly indicates that the CTV coverage of multichannel cylinder is better than the single channel cylinder approach. At the same time the most exposed CTV (D_2cc_) do not show major difference (p=0.003). It is comparing to previous one small variation only occurred. Multichannel approach reduced doses to rectum and bladder. Rectum DVHs analysis showed statistically significant difference between single and multichannel. The p value for 1cc, 2cc and V70 are 0.003, 0.003 and 0.003. On the other hand, bladder DVHs showed large difference between single and multichannel plan yet it is not statistically significant, and the p values for 1cc, 2cc and V80 are 0.012, 0.009 and 0.225. The Table 2 summarized data, clearly indicate that the bladder and rectum dose are reduced, CTV dose is improved by in house multichannel applicator approach. The Figure 4 shows that the dose distribution comparison between single channel and multichannel. It clearly indicates dose to the OAR was reduced in multi channel compare to the single channel. Figure 5 (a) and (b) shows mean and standard Deviation(SD) of CTV D_100_(%), D_95_(%), D_2cc_(Gy) Bladder and D_2cc_(Gy) rectum.

## Discussion

Vaginal cuff brachytherapy is the standard treatment option for endometrial cancer over the decade. These studies investigated the new multichannel applicator dose distribution and quantify its capacity to increase the dose to target and decrease the dose to critical structure. For asymmetric geometrical lesion the single-channel applicator was failure to deliver the maximum dose to lesson and fewer doses to organ at risks. There are other techniques to address this problem such as interstitial brachytherapy and multichannel HDR. The main disadvantages with interstitial brachytherapy are the invasiveness of the procedure; need of the proficiency in this field, bleeding, pain complication and sometime patients need to stay long in hospital (Aime and Gloia, 2014). There is 7% chance of wound infections, abscesses, and fat necrosis in interstitial brachytherapy.

Richardson et al., (2010) concluded that around the cylinder rarely air pocket was rarely created, due to that radiation dose is reduced to target. The multichannel applicator was made up of PMMA material and 3 cm diameter if the applicator was not fitted properly in vagina or nature of applicator, there was always possibility of air pocket between applicator and vaginal wall. In our study we have not selected the case which contains the air pocket. Only one case we found air pocket around the cylinder. Symon et al., (2006) concluded that there is no advantage of re-planning for each fraction, so we analyzes only first fraction. Most commonly used commercial multichannel applicator are Miami 7-channels vaginal applicator (Varian Medical Systems, Palo Alto, CA, USA) and the Capri 13 -channel applicator (Varian Medical Systems, Charlottesville, VA) however this applicators are too costly so most of the developing county still using single channel.

The multichannel generally required longer planning time, around mean 120 minutes which higher than the single channel planning time. The result of preliminary evaluation of the in-house multichannel applicator shows that the bladder and rectum dose was reduced without compromising dose to target. Briefly discussing, the aim of radiotherapy is to maximize the dose received by a specific region of interest while minimizing the dose to the surrounding normal structures and our in-house multichannel applicator reduced the dose to critical structures. A recent ABS survey by Small et al. (Small et al., 2012) indicated that more than two third of all respondents performed HDR-VBT, and more than 90% of the users selected a single channel vaginal cylinder showing an increasing trend for the simple HDR-VBT.

Our data comparing to Yasir et al., (2014) it is observed that the CTV, rectum and bladder dose of the single channel and multichannel plan for CTV (p=0.008, p=0.004, and p=0003), Rectum (p=0.003, p=0.003, and p=0.003), and Bladder (p=0.012, p=0.009, and p=0.225) respectively. In our study, we got the CTV, rectum, and bladder dose of the single channel and multichannel plan for CTV (p=0.008), D_100_(%) of CTV (p=0.004) and 2cc (Gy) of CTV (p=0.003). The p value for 1cc (Gy), 2cc (Gy) and V70 (%) are 0.003, 0.003 and 0.003 for rectum. It is comparing to published one, we got expected p values. 

The in-house multichannel applicator made of PMMA is reusable and can be sterilized for several times in between fractions and for multiple patients. Our applicator is less expensive as compared to the commercially available muti channel applicators hence it is cost effective. The authors conclude that multichannel applicator design improves the dosimetry over single channel applicators by providing superior coverage of tumor volumes and reducing the organ at risk.
